# In Memoriam Martin S. Wolfe (1935–2017)

**DOI:** 10.4269/ajtmh.17obit1

**Published:** 2017-09-07

**Authors:** Elaine Jong

With the death of longstanding ASTMH member Martin S. Wolfe, MD, DCMT, FACP, FASTMH, on June 15, 2017, clinicians practicing clinical tropical medicine and traveler’s health have lost a beloved friend and leader. In a career that spanned over five decades, Marty saw more tropical and travel medicine than most. With his unparalleled clinical experience, intellectual rigor, keen insight, and affable personality, Marty guided and mentored countless scientists and clinicians from all over the world. Marty’s unusual clinical case presentations and vibrant discussions always drew a full house at national and international conferences. He was recognized as the de facto leader of the “Clinical Group” years before the American Committee on Clinical Tropical Medicine and Traveler’s Health (ACCTMTH) was formally organized with Marty appropriately serving as its first president. For his teaching and mentorship in tropical medicine, the ASTMH awarded Marty the Ben Kean Medal in 1998, and named him a Fellow of the ASTMH in 2013. Marty’s many achievements in the fields of travel and tropical medicine earned him the nickname “The father of travel medicine.”

Marty was a native of Scranton, Pennsylvania, the only son of a tavern owner and a devoted mother. He was the first in his family to attend college, completing his undergraduate studies at Cornell University and his medical degree at Cornell University Medical College. While in medical school, his future was greatly influenced by the late Benjamin H. Kean, MD, a pioneer in the nascent field of travel medicine who encouraged Marty to learn more about tropical diseases: “…go out and do some field work. Get your hands dirty.” Thus, the seed of his future career was planted. Marty’s personal life also took a turn around this time after he met and fell in love with a young Danish woman, Lise-Lotte Brunes, while traveling in Europe on his way to Russia. She would become his wife of 55 years, mother to his three children and life-long partner.

After completing his internship at George Washington University Hospital, Marty and Lotte moved to Accra, Ghana, where he spent 2 years as a US Public Health Service Officer attached to the NIH West Africa Research Laboratory studying schistosomiasis. He returned to Cornell to complete 2 years of internal medicine residency and then spent a year at the London School of Hygiene and Tropical Medicine as a Rockefeller Foundation Fellow where he received a Diploma in Clinical Medicine of the Tropics. His travels next took Marty and his young family to Lahore, Pakistan, for 2 years. He conducted research at the University of Maryland International Center for Medical Research Training (ICMRT) identifying the distribution of Bancroftian filariasis in what is now Bangladesh. He then returned to the US, and settled in Washington, DC, where he worked for the Department of State as the Tropical Medicine expert. In this role, he assisted thousands of State Department personnel who presented with a great diversity of tropical and travel-related diseases, and he consulted and visited with US embassy medical staff posted around the globe. While at the Department of State, he traveled extensively with Secretary of State Henry Kissinger on diplomatic missions throughout the world. Marty also offered his expertise as a long-time consultant to the World Bank and the Peace Corps.

In the 1970s, as global travel began to increase, Marty recognized that many travelers needed specialized care both before and after travel. Based on his years of prior travel, academic work, and knowledge of clinical tropical medicine, he founded the Washington, DC, area’s first private medical practice dedicated to travelers, Traveler’s Medical Service, and the Parasitology Laboratory of Washington. Despite his many activities, Marty always found time to respond to a colleague’s telephone call asking for help and his expert advice. He had great enthusiasm for solving diagnostic conundrums and was affectionately known as “The Bug Man” for his ability to diagnose and treat even the most obscure travel and exotic diseases.

Throughout his career, Marty also influenced many as a Clinical Professor of Medicine at Georgetown University and George Washington University School of Medicine where he lectured extensively and mentored innumerable clinicians. The author of more than a hundred academic papers, textbook chapters, and monographs in the fields of travel and tropical medicine, Marty worked on two special projects that were dear to him. During the 1980s and 1990s, Marty wrote and edited several editions of the ASTMH publication “Health Hints for the Tropics,” one of the earliest guidebooks on pretravel care for international travelers. Contributing authors were recruited from among members of the Clinical Group. The other was the creation of the CIBA Pharmaceutical Company’s Clinical Symposia edition on “Diseases of International Travel” published in 1985. Marty authored the text and worked very closely with the late physician-artist Frank H. Netter, MD, who created unique images in full color to illustrate the teaching points. This beautifully illustrated and informative booklet was widely distributed to US physicians and greatly contributed to the expanding awareness and importance of travel and tropical medicine among clinicians at that time. Much of the text and medical illustrations created for the original edition of “Diseases of International Travel” were updated and republished with Marty’s active participation in the book “Netter’s Infectious Diseases” published in 2012.

At the annual meetings of the ASTMH, Marty always enjoyed impromptu and lively conversations with colleagues and friends from around the world in the hallways in between scientific sessions. He derived immense pleasure in developing new friendships with clinicians similarly intrigued by exotic diseases, and he had a curiosity about the background and culture of the people he met and the places he visited in the US and abroad. His varied interests and scholarship were evidenced by the eclectic mix of books on history and culture in his personal library. A popular invited lecturer at national conferences, during his free time he would try to visit a local synagogue, attend a baseball game (in any city that had a local or national team), and do some strolling about in the city to observe and try to understand the character of the place. He would often find willing colleagues to join him for these urban adventures that usually included sampling the local culinary flavors.

Marty retired from the practice of medicine in 2015 but enjoyed staying current and engaged, with books and journals ever present, while enjoying time with family, including his seven grandchildren. He was very proud that his passion for travel and tropical medicine was shared by two of his children. Following in his footsteps, Traveler’s Medical Service of Washington is now directed by his son David P. Wolfe, MD. Its New York City affiliate, Traveler’s Medical Service of New York, was founded and is directed by his daughter Rebecca Wolfe Acosta, RN, MPH, and son-in-law, Alberto M. Acosta, MD, PhD. Marty’s legacy and many contributions to the field of travel and tropical medicine are now a part of the foundation that many clinicians rely on in practice; however, for all of those in the Society and beyond who knew and worked with him, his kind, keen and reassuring presence will be greatly missed.

**Figure:**
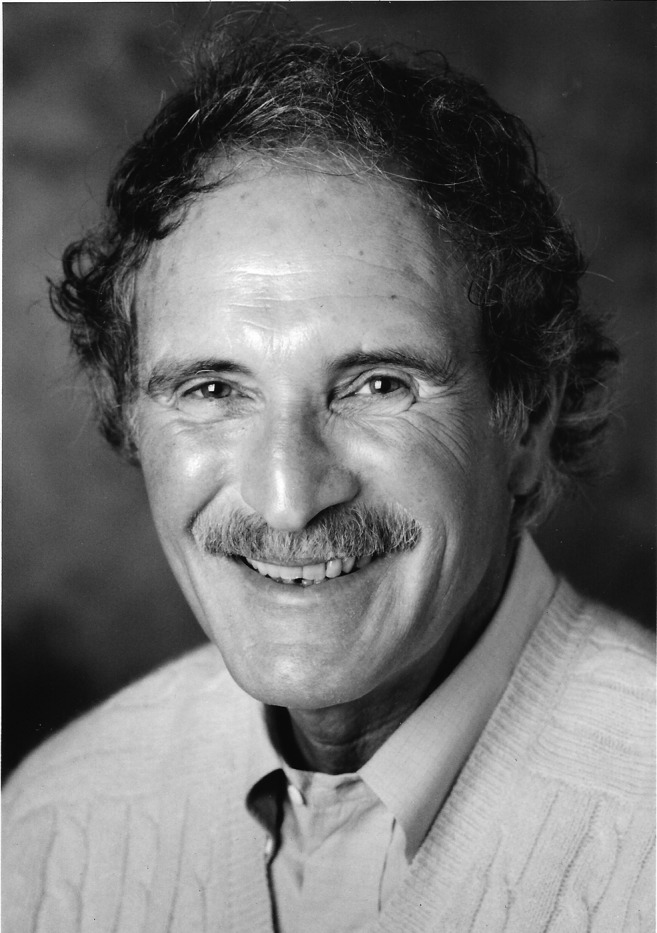
**Martin S. Wolfe (April 9, 1935–June 15, 2017)**

**Figure:**
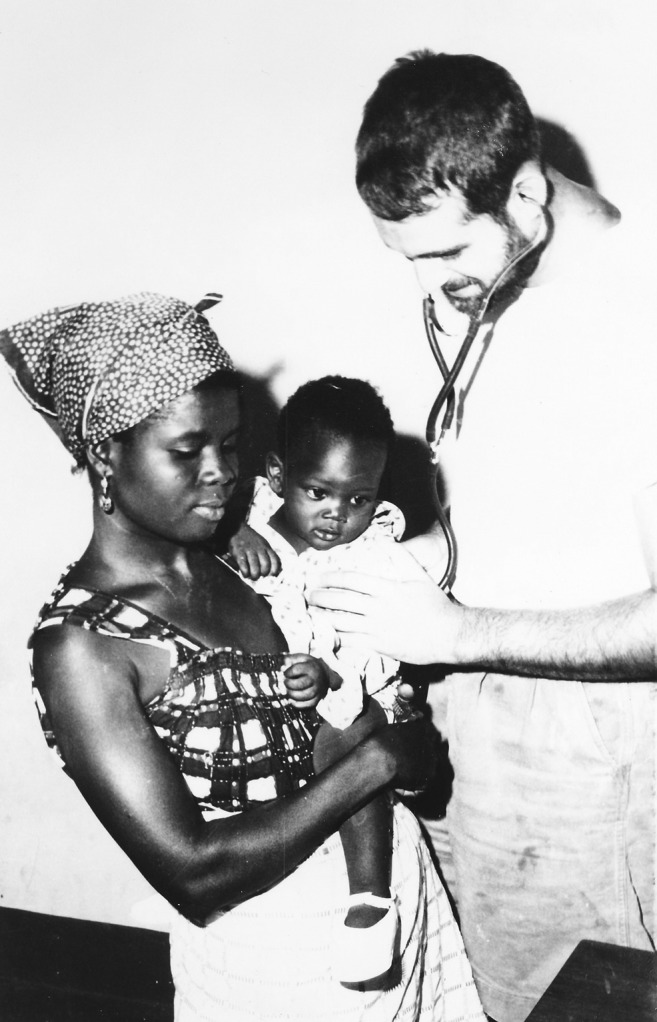
**Dr. Martin S. Wolfe examining a child in Ghana c.1963.**

**Figure:**
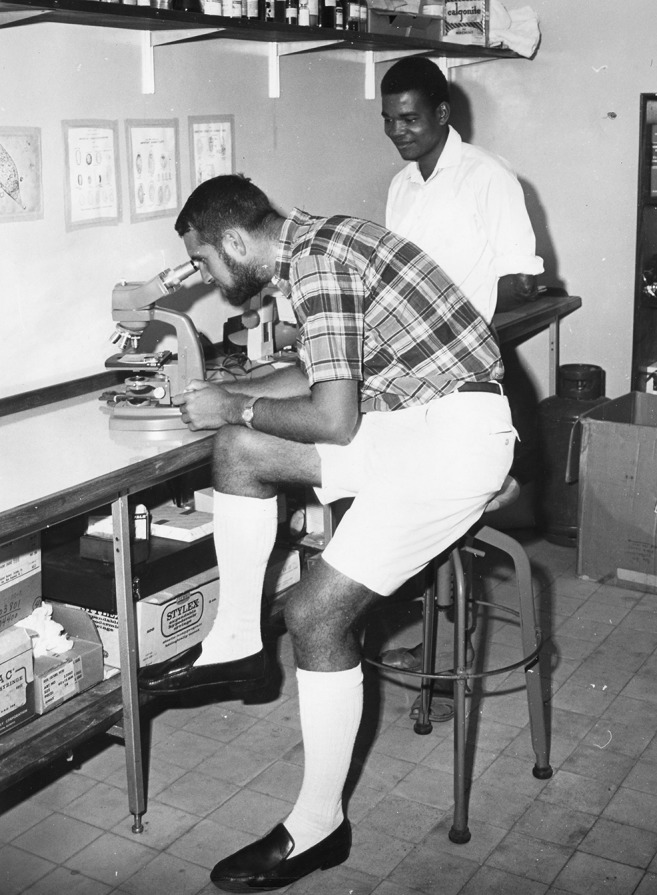
**Dr. Martin S. Wolfe performing microscopic examination of a clinical specimen in Pakistan in the 1960s.**

